# Genome sequences of clinical EHEC isolates from Germany’s large 2025 O45:H2 outbreak, revealing multidrug resistance and unusual strain diversity

**DOI:** 10.1128/mra.00231-26

**Published:** 2026-08-26

**Authors:** Christina Lang, Angelika Fruth, Somayyeh Sedaghatjoo, Valentina Galeone, Antje Flieger

**Affiliations:** 1 Robert Koch Institute, FG 11 Enteropathogenic Bacteria and Legionella, National Reference Centre for Salmonella and Other Enteric Bacterial Pathogens, Wernigerode, Germany; 2 Robert Koch Institute, MF1 Genome Competence Center, Berlin, Germany; University of Pittsburgh School of Medicine, Pittsburgh, Pennsylvania, USA

**Keywords:** EHEC, O45:H2, outbreak

## Abstract

We here present the long-read and short-read genome sequences of 10 representative clinical enterohemorrhagic *Escherichia coli* isolates from the 2025 O45:H2 outbreak in Germany.

## ANNOUNCEMENT

From late August to October 2025, Germany reported a large outbreak of enterohemorrhagic *Escherichia coli* (EHEC)-associated illness, including diarrhea and hemolytic uremic syndrome (HUS). According to the outbreak case definition, more than 400 cases were linked to the event. In total, 62 patients—predominantly children—developed HUS, and three individuals died, underscoring the high virulence of the outbreak strain (1).

The National Reference Centre for *Salmonella* and other Enteric Bacterial Pathogens (NRC) identified the outbreak strain as EHEC serovar O45:H2. The strain harbored the virulence genes *stx2*a and *eaeA*-xi and belonged to multilocus sequence type ST301 (2). Phenotypic testing revealed multidrug resistance to ampicillin, chloramphenicol, kanamycin, nalidixic acid, trimethoprim/sulfamethoxazole, trimethoprim, and tetracycline (2). More than 100 isolates were whole-genome sequenced and assigned to the outbreak cluster using core genome MLST (cgMLST) (3). Here, we present data for 10 representative outbreak isolates sequenced using short-read technology, three of which were additionally subjected to long-read sequencing (Table 1).

**TABLE 1:** Key characteristics of the EHEC O45:H11 stx2a pos and eaeA pos clinical isolates genome sequenced in this study^*b*^

Isolate ID	25-06685	25-07233	25-07338	25-07716	25-07136	25-07232	25-07774	25-08676	25-08677	25-09477-1
GenBank accession no.	JBTRAT000000000/SRR36506198	JBTRIA000000000/SRR36506195	JBTWMM000000000/SRR36506194	JBXVFL000000000/SRR36506193	JBXVFN000000000/SRR36506197	JBXVFM000000000/SRR36506196	JBXVFK000000000/SRR36506192	JBXVFJ000000000/SRR36506191	JBXVFI000000000/SRR36506190	JBXVFH000000000/SRR36506189
CT^*a*^	66411	66411	66411	66682	66839	66840	67134	67979	67980	68756
Long-read sequencing method (flow cell)	GridION (FLO-MIN114)	GridION (FLO-MIN114)	GridION (FLO-MIN114)	n.a.	n.a.	n.a.	n.a.	n.a.	n.a.	n.a.
Short-read sequencing method	Illumina NextSeq (2 × 150 bp)	Illumina NextSeq (2 × 150 bp)	Illumina NextSeq (2 × 150 bp)	Illumina NextSeq (2 × 150 bp)	Illumina NextSeq (2 × 150 bp)	Illumina NextSeq (2 × 150 bp)	Illumina NextSeq (2 × 150 bp)	Illumina NextSeq (2 × 150 bp)	Illumina NextSeq (2 × 150 bp)	Illumina NextSeq (2 × 150 bp)
Library type	ONT ligation	ONT ligation	ONT ligation	Nextera XT	Nextera XT	Nextera XT	Nextera XT	Nextera XT	Nextera XT	Nextera XT
Instrument	GridION/NX4	GridION/NX4	GridION/NX4	NX4	NX4	NX4	NX4	NX4	NX4	NX4
Genome size (bp)	5,403,850	5,403,850	5,403,850	n.a.	n.a.	n.a.	n.a.	n.a.	n.a.	n.a.
Plasmid 1 (bp)	157,778	157,778	157,778	n.a.	n.a.	n.a.	n.a.	n.a.	n.a.	n.a.
Plasmid 2 (bp)	97,727	97,727	97,727	n.a.	n.a.	n.a.	n.a.	n.a.	n.a.	n.a.
Plasmid 3 (bp)	97,648	97,648	97,648	n.a.	n.a.	n.a.	n.a.	n.a.	n.a.	n.a.
Plasmid 4 (bp)	5,114	5,114	5,114	n.a.	n.a.	n.a.	n.a.	n.a.	n.a.	n.a.
No. of protein-coding genes	5,709	5,704	5,709	n.a.	n.a.	n.a.	n.a.	n.a.	n.a.	n.a.
No. of rRNA operons	22	22	22	n.a.	n.a.	n.a.	n.a.	n.a.	n.a.	n.a.
No. of tRNA genes	101	101	101	n.a.	n.a.	n.a.	n.a.	n.a.	n.a.	n.a.
No. of long reads	705,534	678,947	566,314	n.a.	n.a.	n.a.	n.a.	n.a.	n.a.	n.a.
N50 long reads/assembly contigs	10,284	10,146	9,831	87,678	87,568	71,602	73,973	96,381	100,988	100,118
No. of contigs	5	5	5	435	423	458	443	380	381	418
GC content long reads/assembly contigs (%)	50.47	50.49	50.41	50.37	50.38	50.4	50.37	50.38	50.39	50.38
GC content short reads (%)	50.29	50.71	51.05	50.83	50.95	50.99	50.82	50.95	50.83	50.29
No. of short reads	2,898,066	17,562,638	2,715,508	8,607,640	3,677,256	3,000,228	3,457,892	3,972,274	4,036,490	3,291,392

^*a*^ CT, complex type according to 2,513 cgMLST genes; n.a., not applicable.

^*b*^ All isolates were obtained from stool swabs, belonging to serotype O45:H2, and were assigned to sequence type ST301.

EHEC single colonies recovered on enterohemolysin agar were cultured overnight at 37°C in 3 mL Luria–Bertani broth prior to DNA extraction (4). Genomic DNA for long-read sequencing was extracted using the Qiagen Genomic-tip 100 and Genomic DNA buffer set (Qiagen) according to the manufacturer’s instructions, while DNA for short-read sequencing was prepared by glass-bead extraction (5). DNA quality was assessed using a NanoDrop spectrophotometer, and concentrations were analyzed using a Qubit fluorometer (Thermo Fisher Scientific).

Sequencing was performed on Illumina and Oxford Nanopore platforms. Illumina libraries were prepared from 1 ng DNA using the Nextera XT DNA Library Preparation Kit and sequenced on a NextSeq 2000 (2 × 150 bp paired-end). Nanopore libraries were prepared using the Native Barcoding Kit (SQK-NBD114.24) and sequenced on a GridION with an R10.4.1 flow cell (FLO-MIN114). FastQC v0.12.1 was used for Illumina read data quality control (6). ONT read quality was assessed using NanoPlot v1.46.1 (7). Nanopore base calling and demultiplexing were performed with Dorado v1.1.1 (8); assemblies (including genome and plasmid ring closures) were generated with Autocycler v0.5.2 (9) and polished with Illumina reads using Polypolish v0.6.0 (10). Annotation was performed with NCBI PGAP (11). Ridom SeqSphere+ (v10.5.4) was used for SKESA assembly (12), cgMLST cluster analysis, and assignment of complex types (CTs) and sequence types (13). Sequence statistics were generated using SeqKit (v2.12.0) (14). All software was run with default parameters.

The outbreak isolates outlined here belonged to eight different cgMLST CTs, indicating unusual genetic diversity within the outbreak cluster (Table 1). Specifically, three isolates belonged to the prevalent CT 66411 and were analyzed using both short-read and long-read sequencing. Our analysis revealed chromosomal antimicrobial resistance genes (*blaEC*, *dfrA36*, and *sul2*) and four plasmids, including one carrying resistance genes (*aph(3′)-Ia*, *blaTEM-1*, *catA1*, and *tet(A*)) (Table 1). Short-read sequencing was performed for seven outbreak isolates representing seven further CTs (Table 1).

Overall, these genome data characterize a highly pathogenic, multidrug-resistant, and genetically diverse O45:H2 EHEC outbreak strain causing substantial morbidity, including severe HUS and fatalities, and provide a foundation for future outbreak strain detection and source tracing.

## Data Availability

The closed genome sequences, plasmids, assemblies, and associated raw data were deposited at NCBI under BioProject PRJNA1390743. All accession numbers are listed in Table 1.
